# COVID‐19 Infection Enhances Susceptibility to Oxidative Stress–Induced Parkinsonism

**DOI:** 10.1002/mds.29116

**Published:** 2022-06-09

**Authors:** Richard J. Smeyne, Jeffrey B. Eells, Debotri Chatterjee, Matthew Byrne, Shaw M. Akula, Srinivas Sriramula, Dorcas P. O'Rourke, Peter Schmidt

**Affiliations:** ^1^ Department of Neurosciences Thomas Jefferson University Vickie and Jack Farber Institute for Neuroscience Philadelphia Pennsylvania USA; ^2^ Department of Anatomy and Cell Biology Brody School of Medicine East Carolina University Greenville North Carolina USA; ^3^ Department of Microbiology & Immunology Brody School of Medicine East Carolina University Greenville North Carolina USA; ^4^ Department of Pharmacology and Toxicology Brody School of Medicine East Carolina University Greenville North Carolina USA; ^5^ Department of Comparative Medicine Brody School of Medicine East Carolina University Greenville North Carolina USA; ^6^ Department of Neurology Grossman School of Medicine New York University New York New York USA

**Keywords:** COVID‐19, MPTP, Parkinson's disease

## Abstract

**Background:**

Viral induction of neurological syndromes has been a concern since parkinsonian‐like features were observed in patients diagnosed with encephalitis lethargica subsequent to the 1918 influenza pandemic. Given the similarities in the systemic responses after severe acute respiratory syndrome coronavirus 2 (SARS‐CoV‐2) infection with those observed after pandemic influenza, there is a question whether a similar syndrome of postencephalic parkinsonism could follow coronavirus disease 2019 infection.

**Objective:**

The goal of this study was to determine whether prior infection with SARS‐CoV‐2 increased sensitivity to a mitochondrial toxin known to induce parkinsonism.

**Methods:**

K18‐hACE2 mice were infected with SARS‐CoV‐2 to induce mild‐to‐moderate disease. After 38 days of recovery, mice were administered a non‐lesion‐inducing dose of the parkinsonian toxin 1‐methyl‐4‐phenyl‐1,2,3,6‐tetrahydropyridine (MPTP) and euthanized 7 days later. Subsequent neuroinflammation and substantia nigra pars compacta (SNpc) dopaminergic (DA) neuron loss were determined and compared with SARS‐CoV‐2 or MPTP alone.

**Results:**

K18‐hACE2 mice infected with SARS‐CoV‐2 or MPTP showed no SNpc DA neuron loss after MPTP. In mice infected and recovered from SARS‐CoV‐2 infection, MPTP induced a 23% or 19% greater loss of SNpc DA neurons than SARS‐CoV‐2 or MPTP, respectively (*P* < 0.05). Examination of microglial activation showed a significant increase in the number of activated microglia in both the SNpc and striatum of the SARS‐CoV‐2 + MPTP group compared with SARS‐CoV‐2 or MPTP alone.

**Conclusions:**

Our observations have important implications for long‐term public health, given the number of people who have survived SARS‐CoV‐2 infection, as well as for future public policy regarding infection mitigation. However, it will be critical to determine whether other agents known to increase risk for PD also have synergistic effects with SARS‐CoV‐2 and are abrogated by vaccination. © 2022 The Authors. *Movement Disorders* published by Wiley Periodicals LLC on behalf of International Parkinson and Movement Disorder Society

## Graphical abstract

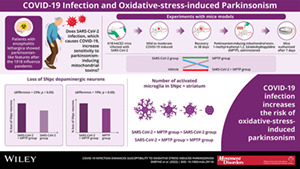

July Infographic : COVID‐19 Infection Enhances Susceptibility to Oxidative Stress–Induced Parkinsonism

Neurological sequalae subsequent to viral infection have been reported since von Economo's^1^ description of a postinfluenza encephalopathy, including aspects of parkinsonism, that was reported to the Vienna neurological society in 1917. These observations were consistent with the postencephalic symptoms that followed the 1918 H1N1 (Spanish) influenza pandemic and persisted for about 10 years subsequent to its zenith.^2^, ^3^ Although the 1918 influenza was thought to be particularly virulent and its sequalae particularly devastating, it is not the only viral outbreak that has been linked to postencephalic parkinsonian symptoms.^4^ The mechanism for this linkage is likely a viral affinity for the highly vascularized midbrain catecholaminergic neurons in the substantia nigra and locus coeruleus^5^ that are lost in Parkinson's disease (PD). Mechanisms of indirect action after viral infection, such as effects of inflammatory cytokines or glia activation, have been demonstrated previously.^6^, ^7^

In 2019, a novel coronavirus outbreak was reported in China and in the ensuing pandemic, nearly 516 million cases have been reported worldwide.^8^ The virus, severe acute respiratory syndrome coronavirus 2 (SARS‐CoV‐2), is a large, enveloped, nonsegmented, positive‐sense RNA virus.^9^ Like its related family members, SARS‐CoV‐1 and Middle East respiratory syndrome, it predominantly presents as a respiratory illness^10^; however, a number of other organ systems,^11^ including the nervous system, are also severely affected.^12^ Relating specifically to SARS‐CoV‐2, it is unclear whether these effects are direct, based on the virus's ability to enter the brain,^13^ or whether they arise via a peripheral mechanism, such as an induction of a cytokine storm that induces neurological changes in the peripheral immune system that then transmits its signals to the brain.^14^

Given the long history of viral infections inducing basal ganglia disease,^4^ combined with the scale and scope of the coronavirus disease 2019 (COVID‐19) pandemic, it is incumbent to explore whether there might be an increased risk for similar neurological sequelae among individuals recovered from COVID‐19. Recent reports have empirically associated SARS‐CoV‐2 infection with development of clinical parkinsonism.^15^, ^16^, ^17^ To experimentally test the hypothesis that prior infection with SARS‐CoV‐2 could directly increase the risk for parkinsonism through viral mechanisms identified previously, we evaluated nigrostriatal degeneration in a preclinical mouse model where genetically tailored susceptible animals, engineered to express the human angiotensin‐converting enzyme 2 (hACE2) receptor,^18^ were infected with SARS‐CoV‐2 (strain USA‐1) and allowed to recover. The hACE2 mouse model for studies of central nervous system (CNS) effects of the disease, including on the blood–brain barrier (BBB), were previously studied and found to be illustrative for human infection/disease.^19^, ^20^ Thirty‐eight days after the animals recovered from the viral infection, we challenged them with subtoxic levels of a mitochondrial toxin, 1‐methyl‐4‐phenyl‐1,2,3,6‐tetrahydropyridine (MPTP), a chemical that is known to block complex I and IV of the electron transport chain and induce some of the characteristic pathologies seen in PD.^21^ We found that animals that recovered from SARS‐CoV‐2 infection were more susceptible to the parkinsonian effects of nonlethal levels of MPTP than mice infected with SARS‐CoV‐2 or administered MPTP alone. This preclinical study suggests that infection with SARS‐CoV‐2 is likely a predisposing risk factor for later development of PD.

## Materials and Methods

All the work pertaining to the use of SARS‐CoV‐2 (isolate USA‐WA1/2020; bei RESOURCES, Manassas, VA, USA) was performed at BSL‐3 levels at East Carolina University (Greenville, NC, USA). Virus was propagated in Vero cells using a 10–30% sucrose gradient in an ultracentrifuge, and the yield was titrated using Reed and Muench calculations.^22^, ^23^, ^24^ Viral studies were approved by the Office of Prospective Health/Biological Safety for the use of SARS‐CoV‐2 (registration number 20–01).

### SARS‐CoV‐2 Infection

**FIG 1:**
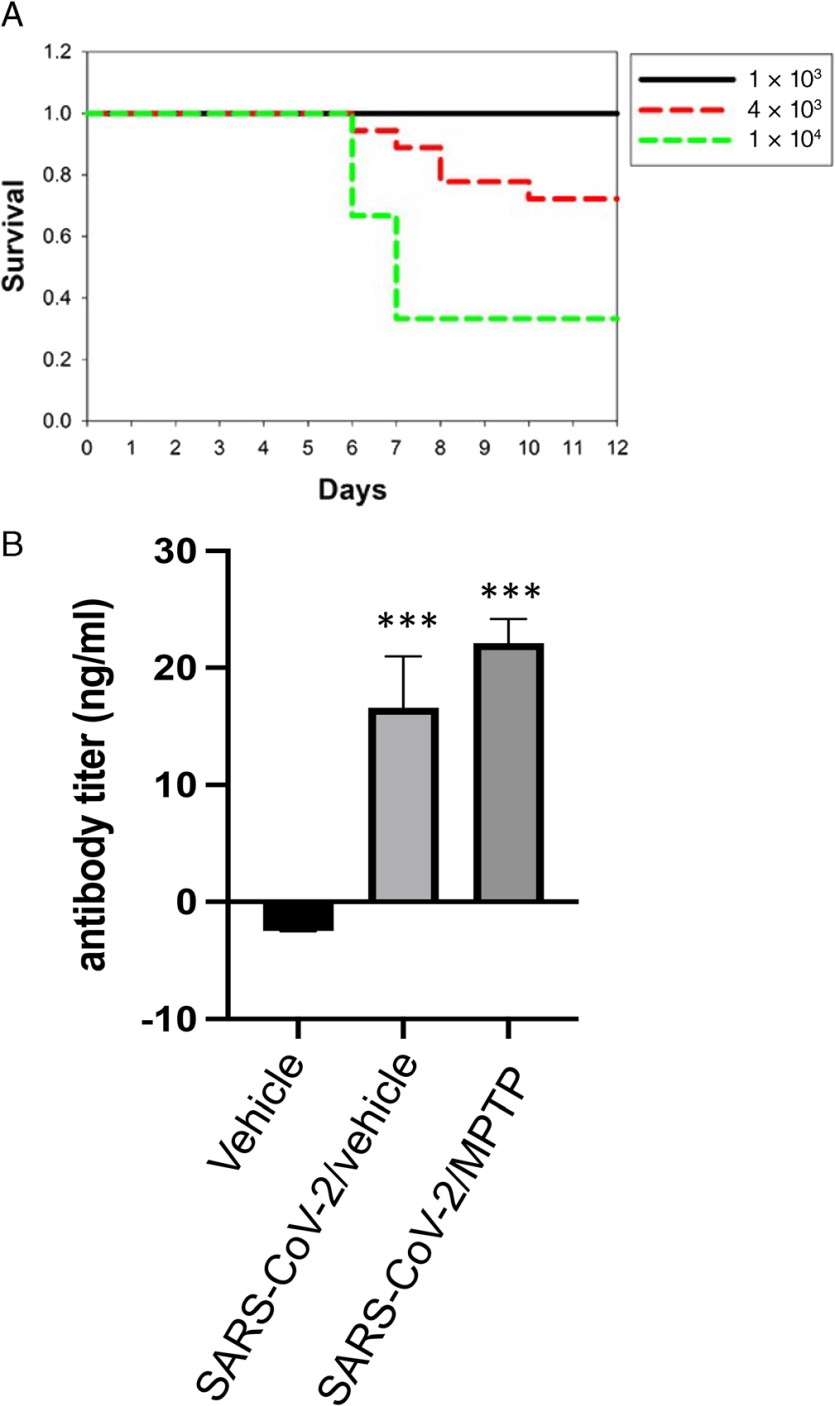
Determination of severe acute respiratory syndrome coronavirus 2 (SARS‐CoV‐2) dose and antibody titer. (**A**) Kaplan–Meier survival curve of male mice infected with three different titers of SARS‐CoV‐2 (USA‐1). High dose (1 × 10^3^ TCID_50_): n = 3; intermediate dose (4 × 10^3^ TCID_50_): n = 3; low dose (1 × 10^4^ TCID_50_): n = 3. (**B**) Antibody titers in mice infected 45 days before testing. Vehicle: n = 10; SARS‐Co‐V‐2/vehicle: n = 7; SARS‐CoV‐2 + 1‐methyl‐4‐phenyl‐1,2,3,6‐tetrahydropyridine (MPTP): n = 6. ***P < 0.001 compared with control (vehicle) group. Statistical analysis was performed using analysis of variance with post hoc tests (Tukey) (Prism 9.0; GraphPad Software) if overall significance was achieved. [Color figure can be viewed at wileyonlinelibrary.com]

Working under an Institutional Animal Care and Use Committee–approved protocol (AUP#A209) in a fully Association for Assessment and Accreditation of Laboratory Animal Care–accredited facility, 6‐ to 8‐month‐old SARS‐CoV‐2–susceptible mice [B6.Cg‐Tg(K18‐ACE2)2Prlmn/J, Strain #034860, also known as hACE2 mice; Jackson Labs, Bar Harbor, ME] were randomly assigned to a dose‐finding study and then four study arms. Approximately equal numbers of male and female mice were used in each study. Mice were anesthetized with 3% isoflurane for infection. First, nine male mice were tested at three doses of virus (three mice each at 10,^3^ 4 × 10^3^, and 10^4^ Median Tissue Culture Infectious Dose (TCID_50_)). In the low dose, all survived. At the high dose, two of three mice were euthanized because of severe symptoms. At the intermediate dose, one of three mice was euthanized; thus, the 4 × 10^3^ TCID_50_ dose was selected as the optimal dose (Fig. 1) with mortality similar to the viral load in humans correlated to mortality.^25^ Using 4 × 10^3^ TCID_50_ in 25 μl saline, an additional 16 mice (7 male and 9 female for a final total of 9 male and 9 female mice) were infected intranasally with 4 × 10^3^ TCID_50_ SARS‐CoV‐2 (divided equally into 12.5 μl in each nostril) and 12 mice (6 male and 6 female) were subjected to a sham procedure (12.5 μl saline into each nostril). Animals were observed for signs of infection, decreases in body weight, alterations in body temperature, lack of grooming, hunched posture, rapid respiration, lethargy, and mortality and were managed under the supervision of an American College of Laboratory Animal Medicine board–certified laboratory animal veterinarian (D.P.O.). Continuous monitoring of animal temperature and activity was provided by an RFID (radiofrequency identification) microchip and sensors using the UID Mouse Matrix and UID Temperature Microchips (https://www.uidevices.com). To confirm infection in mice, we collected blood via cardiac puncture of each animal before perfusion, placed it in KE EDTA tubes, and isolated plasma. Antibody titers were measured in plasma using the Mouse Anti‐SARS‐CoV‐2 IgG Antibody ELISA Kit (DEIASL240) from CD Creative Diagnostics (NY, NY) according to the manufacturer's instructions.

Five of the 18 mice, 3 male and 2 female mice, infected at 4 × 10^3^ TCID_50_ were euthanized because of severe symptoms, leaving 13 surviving animals. Thirty‐eight days after the infection procedure, six SARS‐CoV‐2 and six sham‐treated mice (three male and three female) were challenged with MPTP, a mitochondrial stressor known to induce some of the characteristic pathologies of PD that has been previously used in similar studies.^26^, ^27^ For the final analysis, all data from male and female mice were combined because we found no significant differences in males versus females in regard to response to sublethal doses of MPTP (*P* < 0.47) or from SARS‐CoV‐2 (*P* < 0.43) infection. In addition, historical data in the Smeyne lab of more than 250 animals using the acute paradigm of MPTP have shown no sex differences in regard to SNpc dopaminergic (DA) neuron loss (R.J.S., personal communication). In this article, we used a subtoxic dose of MPTP (10 mg/kg × 4 at 2‐hour intervals, intraperitoneally) that has been shown to induce a small inflammatory response, but no SNpc DA neuron death.^27^ To confirm that the mice used were infected with SARS‐CoV‐2, we injected seven mice recovered from SARS‐CoV‐2 (four male and three female nice) and six sham‐treated (three male and three female) mice intraperitoneally with equal volumes of saline.

Forty‐five days after active or sham infection and 7 days after MPTP or saline administration, animals were anesthetized and euthanized by transcardial perfusion with 3% paraformaldehyde.

### Quantitation of SNpc DA Neuron Number and Microglial Number

After perfusion, brains were dissected from the calvaria, postfixed overnight, then dehydrated through graded ethanols, defatted in xylene, and embedded in the coronal plane in paraffin (Paraplast‐Xtra; Fisher Scientific, Waltham, MA, USA). Serial 10‐μm sections (five sections per slide) were cut and all sections mounted onto Superfrost‐Plus slides (Fisher Scientific). Every other slide (sampling at 100‐μm intervals) was immunostained for tyrosine hydroxylase (TH, 1:250; mouse monoclonal, T1299; Sigma‐Aldrich, St. Louis, MO, USA) and ionized calcium‐binding adapter molecule 1 (Iba‐1) to immunolabel microglia (1:200; rabbit polyclonal, 019‐19741; Wako, Richmond, VA, USA)^28^ using a two‐color diaminobenzidine (DAB) protocol.

The total number of SNpc TH‐positive DA neurons (+ Nissl) were estimated by model‐based stereology^29^ using the physical disector (StereoInvetigator; MBF Bioscience, Williston, VT). On average, 40 sections per SNpc were analyzed. We used n = 6 for each experimental group, and these included equal numbers of male and female animals. Based on historical data in our laboratory (>250 mice treated with MPTP), the n = 6 in each group will allow us a power of 0.80 accepting a type I error of 0.05.

Microglia numbers were estimated using design‐based stereology (optical disector; StereoInvestigator; MBF Biosciences, Williston, VT).^30^ Microglia were deemed as “resting” if they contained a small oval Iba‐1–positive cell body that averaged three microns or less in diameter with long, slender processes, and as “activated” when the cell body was increased in size compared with resting microglia and had an irregular shape with shorter and thickened processes.^31^

### Evaluation of Striatal Dopamine Terminals and Microglia Density

After perfusions, brains were postfixed overnight in fresh fixative, cryoprotected in 30% sucrose, and then cryosectioned at 30 μm with all sections collected in 1× phosphate‐buffered saline (PBS). Free‐floating sections were incubated for 30 minutes in 3% H_2_O_2_, washed in PBS‐0.1% bovine serum albumin (BSA), and incubated into a 4% goat or rabbit serum (Vector, Burlingame, CA, USA) in PBS‐1% BSA‐0.1%Triton X‐100 for 1 h, then incubated in primary antibody (rabbit anti‐TH, AB152, 1:5000 [Millipore Darmstadt Germany] or goat anti–Iba‐1, 1:500 [FUJIFILM Wako Chemicals] in PBS‐1% BSA‐0.1% Triton X‐100) overnight at 4°C. Sections were then washed five times in PBS‐0.1% BSA and placed into secondary antibody conjugated to horseradish peroxidase (goat anti‐rabbit IgG or rabbit anti‐goat IgG, 1:500 in PBS‐1% BSA‐0.1% Triton X‐100; Invitrogen, Rockford, IL, USA) for 2 h. Sections were rinsed, incubated in 0.25X ImmPACT DAB solution for 4 minutes, washed and mounted on silanized slides, dehydrated with graded ethanols, defatted in xylene, and coverslipped using Permount. For quantification of DA terminals, images were scanned using the MoticEasyScan with the extended depth of focus mode. TH axon densities were calculated using ImageJ software (DAB measurement tool).

The density of resting and activated microglia, determined using the same criteria as used in the SNpc, was made in the dorsolateral striatum. Density was determined in two sections spaced 100 microns apart. The contour of the dorsolateral striatum was outlined and using StereoInvestigator, the area was calculated. Activated and resting microglia within the contour were counted, and using the area measurement, an average density was determined.

## Results

### Effects of SARS‐CoV‐2 Infection on Animal Morbidity and Mortality

B6.Cg‐Tg(K18‐ACE2)2Prlmn/J (K18‐hACE2) mice were infected with one of three different titers (1 × 10^3^ TCID_50_, 4 × 10^3^ TCID_50_, and 1 × 10^4^ TCID_50_) of SARS‐CoV‐2. After exposure, the mice were examined for signs of infection, including decreased body weight, alterations in body temperature, lack of grooming, hunched posture, rapid respiration, and lethargy. We observed no animal morbidity or mortality in mice infected with 1 × 10^3^ TCID_50_, about 28% in mice infected with 4 × 10^3^ TCID_50_, and 67% in animals infected with 1 × 10^4^ TCID_50_ (Fig. 1A). The animals that died were found deceased in their cage or were euthanized with >20% weight loss and appeared moribund, hypothermic, and/or with milky‐white eye discharge. Animals that survived showed no apparent temperature alterations or loss of blood oxygenation. To ensure that surviving mice in the 4 × 10^3^ TCID_50_ group (n = 13) were infected, we performed antibody titers 45 days after infection. As shown in Figure 1B, no detectable viral titer was measured in animals intranasally administered the saline alone. Both the SARS‐CoV‐2– and SARS‐CoV‐2 + MPTP–treated infected mice had a significantly increased antibody response (F_2,20_ = 29.56; *P* < 0.001) compared with saline‐treated mice, although the response between the SARS‐CoV‐2– and SARS‐CoV‐2 + MPTP–treated infected mouse groups was not significantly different (*P* < 0.337). Based on these data, we examined the effects of subtoxic levels of MPTP on neuroinflammation and SNpc DA neuron death at 4 × 10^3^ TCID_50_.

### Prior Infection with SARS‐CoV‐2 Increases Susceptibility to the DA Toxin, MPTP

**FIG 2:**
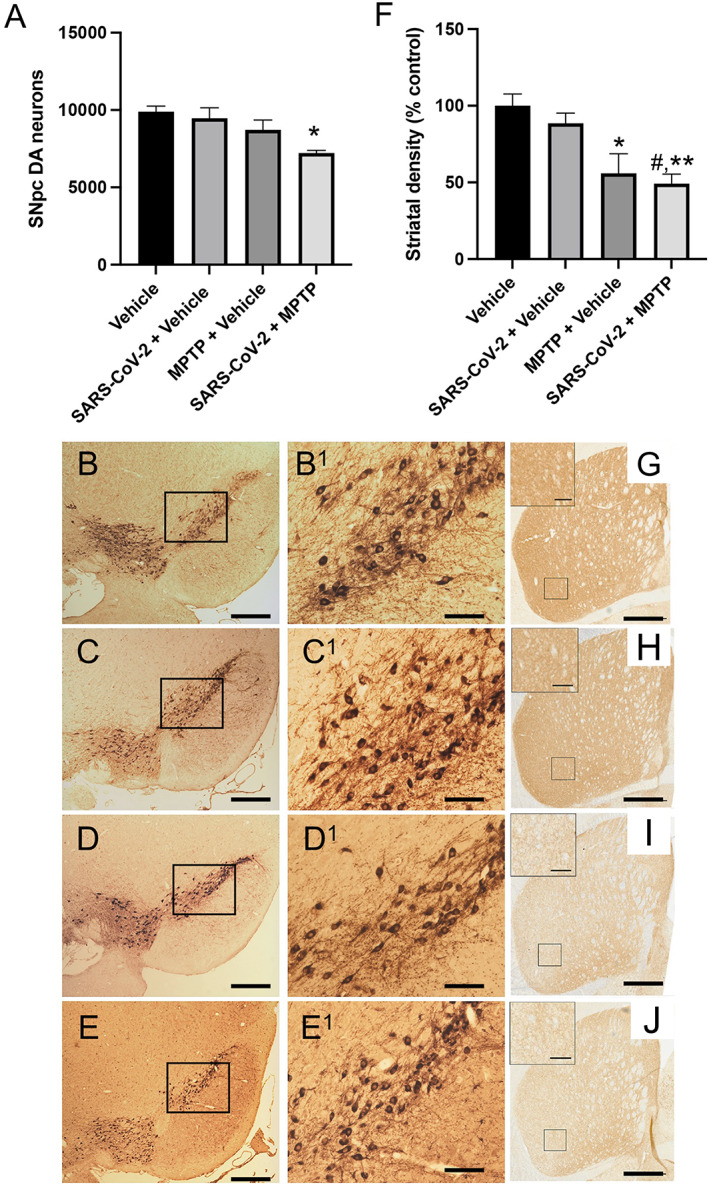
Synergistic effect of substantia nigra pars compacta (SNpc) DA neuron number and terminal field loss induced by severe acute respiratory syndrome coronavirus 2 (SARS‐CoV‐2) with or without 1‐methyl‐4‐phenyl‐1,2,3,6‐tetrahydropyridine (MPTP). (**A**) SNpc DA neuron loss. We observed a significant loss of SNpc DA neurons was measured in the SARS‐CoV‐2 + MPTP group compared with vehicle. (**B**) Low‐power photomicrograph of the SNpc, rostral to the medial longitudinal fasciculus, in vehicle (saline)‐treated mice. (**B** ^**1**^) Higher‐power magnification of the inset in (B). SNpc DA neurons are immunostained with antibodies directed against tyrosine hydroxylase (TH) and then visualized with the chromogen diaminobenzidine (DAB) so that they appear dark brown. (**C**) Low‐power photomicrograph of the SNpc, rostral to the medial longitudinal fasciculus, in SARS‐CoV‐2–treated mice. (**C** ^**1**^) Higher‐power magnification of the inset in (C). (**D**) Low‐power photomicrograph of the SNpc, rostral to the medial longitudinal fasciculus, in 10 mg/kg × 4 MPTP–treated mice. (**D** ^**1**^) Higher‐power magnification of the inset in (D). (**E**) Low‐power photomicrograph of the SNpc, rostral to the medial longitudinal fasciculus, in SARS‐CoV‐2 + MPTP–treated mice. (**E** ^**1**^) Higher‐power magnification of the inset in (E). (**F**) The density of DA neurons. Optical density measurements of TH in the dorsoventral striatum of SARS‐CoV‐2–infected, MPTP‐, and SARS‐CoV‐2 + MPTP–treated mice. Both sublethal MPTP and SARS‐CoV‐2 + MPTP induce a significant phenotypic loss of SNpc DA neuron terminal fields. (**G**) Low‐power photomicrograph of tyrosine hydroxylase–immunostained striatum of the C57BL/6J in vehicle (saline)‐treated mice. Inset: magnification of outlined area in (G). (**H**) Low‐power photomicrograph of tyrosine hydroxylase–immunostained striatum of the C57BL/6J in SARS‐CoV‐2–treated mice. Inset: magnification of outlined area in (H). (**I**) Low‐power photomicrograph of tyrosine hydroxylase–immunostained striatum of the C57BL/6J mice 7 days after 4 × 10 mg/kg MPTP. Inset: magnification of outlined area in (I). (**J**) Low‐power photomicrograph of tyrosine hydroxylase–immunostained striatum of the C57BL/6J mice 38 days after SARS‐CoV‐2 and then 7 days after 4 × 10 mg/kg MPTP. Inset: magnification of outlined area in (J). Scale bars: 150 mm (B–E, G–J); 50 mm (B^1^–E^1^); 20 mm (insets, G–J). *P < 0.05 versus control; **P < 0.01 versus control; ^#^ *P* < 0.05 versus SARS‐CoV‐2. Statistical analysis was performed using analysis of variance with post hoc tests (Tukey) (Prism 9.0; GraphPad Software) if overall significance was achieved. [Color figure can be viewed at wileyonlinelibrary.com]

In this study, we administered a subtoxic dosage of MPTP (10 mg/kg × 4, intraperitoneally every 2 hours) that we have previously shown to induce a small inflammatory response but no SNpc DA neuron death.^27^ We examined the effects of these low levels of MPTP on four groups of mice: (1) sham + vehicle (n = 6), (2) SARS‐CoV‐2 + vehicle (n = 7), (3) sham + MPTP (n = 6), and (4) SARS‐CoV‐2 + MPTP (n = 6). Equal numbers of male and female mice were used, and all mice survived MPTP treatment. We then waited 7 days after MPTP and euthanized mice to assess both SNpc DA neuron and microglia numbers. There was an overall significant difference between groups (F_3,19_ = 4.985; *P* < 0.010). As shown in Figure 2A, no SNpc DA neuron loss was observed after saline, SARS‐CoV‐2, or MPTP in vehicle alone (Fig. 2B–D^1^). However, in mice infected with 4 × 10^3^ TCID_50_ SARS‐CoV‐2 and allowed to recover for 38 days, this concentration (10 mg/kg MPTP × 4) induced a significant 23% (*P* < 0.012) or 19% (*P* < 0.023) greater loss of SNpc DA neurons than SARS‐CoV‐2 or MPTP alone, respectively (Fig. 2A,E,E^1^).

We also evaluated the effect of SARS‐CoV‐2, MPTP, and SARS‐CoV‐2 + MPTP on the SNpc DA neuron terminals in the striatum. We found an overall significance between the four experimental conditions (F_3,20_ = 7.947; *P* < 0.001) (Fig. 2F). Comparison between groups shows that MPTP alone reduces the DA striatal terminal field by 55% compared with vehicle (*P* < 0.012), while the SARS‐CoV‐2 + MPTP induces a reduction rate of 50.1% (*P* < 0.003) (Fig. 2G–J). Unlike the SNpc DA neuron loss, there were no significant differences between the terminal loss after MPTP compared with the SARS‐CoV‐2 + MPTP group. The loss of phenotype in the striatal DA terminals in the MPTP group in excess of neuron loss was not unexpected, because this dichotomy has been previously reported,^32^, ^33^ suggesting that the SNpc DA terminals are more sensitive to oxidative stress than the soma.

Due to the restrictions of animal use in the BL3 facility, we were not able to assess whether the loss of SNpc DA neurons and projections of these neurons to the striatum had any behavioral effect. However, given the relatively small loss of SNpc DA neurons and projections compared with mice given the full acute dose of MPTP (4 × 20 mg/kg × 4), which has not produced chronic behavioral changes, these were not expected.^34^, ^35^, ^36^

### Prior Infection with SARS‐CoV‐2 Increases Neuroinflammation

To examine whether SARS‐CoV‐2 infection induced a neuroinflammatory response, we quantitated total, resting, and activated microglia in the SNpc and resting and activated microglia density in the dorsolateral striatum following vehicle (n = 5), SARS‐CoV‐2 (n = 7), 10 mg/kg × 4 MPTP (n = 6), and SARS‐CoV‐2 + 10 mg/kg MPTP (n = 6).

**FIG 3:**
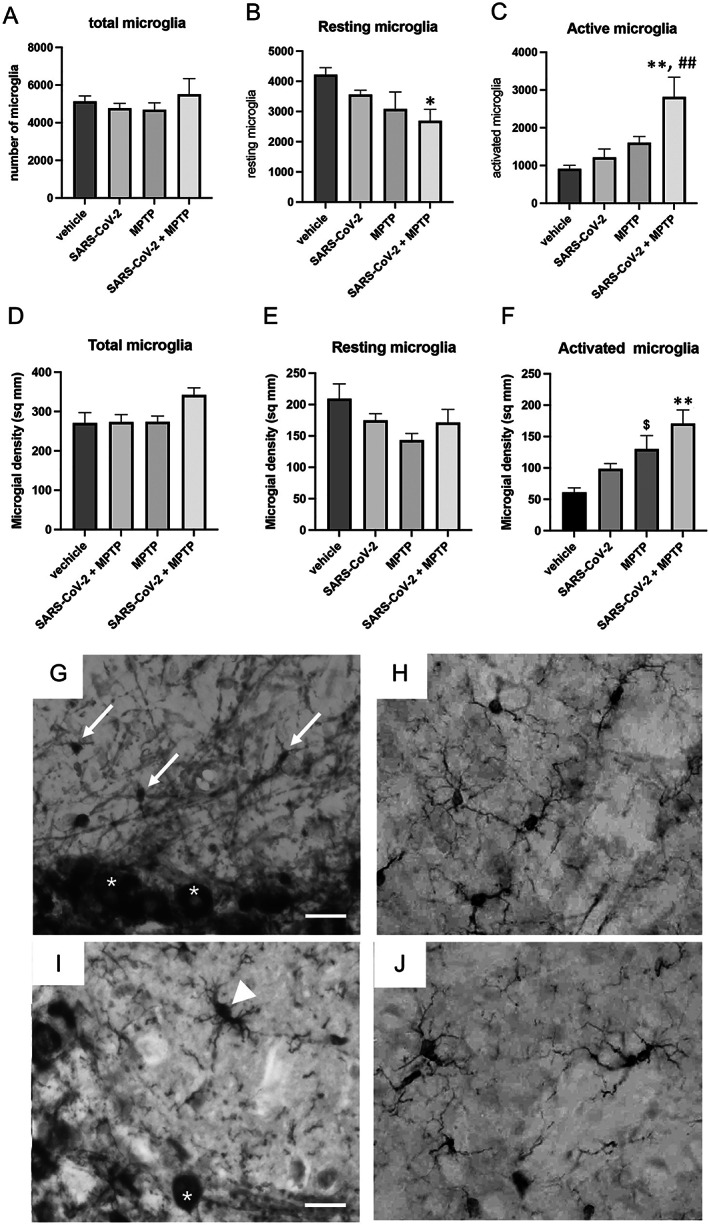
Synergistic effect on neuroinflammation in the substantia nigra pars compacta (SNpc) and dorsolateral striatum by SARS‐CoV‐2 with or without 1‐methyl‐4‐phenyl‐1,2,3,6‐tetrahydropyridine (MPTP). (**A**) Quantitation of total microglia number in the SNpc. (**B**) Quantitation of resting microglia in the SNpc. There is a significant decrease in the number of resting microglia in the SARS‐CoV‐2 + MPTP–treated mice compared with the vehicle‐treated animals. (**C**) Quantitation of active microglia in the SNpc. There is a significant increase in the number of resting microglia in the SARS‐CoV‐2 + MPTP–treated mice compared with both vehicle‐treated and SARS‐CoV‐2–treated animals. (**D**) Quantitation of total microglia density in the dorsolateral striatum. (**E**) Quantitation of resting microglia density in the dorsolateral striatum. (**F**) Quantitation of active microglia density in the dorsolateral striatum. There is a significant increase in the number of resting microglia in the SARS‐CoV‐2 + MPTP–treated mice compared with vehicle‐treated, MPTP alone, and SARS‐CoV‐2–treated animals. MPTP alone induces a significant increase in the density of activated microglia compared with vehicle and SARS‐CoV‐2 alone. **P < 0.01 compared with vehicle or SARS‐CoV‐2 alone; ^##^ *P* < 0.01 compared with vehicle or MPTP alone; ^$^ *P* < 0.05 compared with vehicle. (**G**) Appearance of resting microglia in the SNpc. We classified resting microglia as having soma less than 3 mm in diameter with fine processes emanating from the soma (*white arrows*). For size comparison, the SNpc DA neurons are marked by white asterisks. (**H**) Appearance of resting microglia in the dorsolateral striatum. We classified resting microglia as having soma less than 3 mm in diameter with fine processes emanating from the soma (*white arrows*). For size comparison, the SNpc DA neurons are marked by white asterisks. (**I**) Appearance of activated microglia in the SNpc. We classified activated microglia as having soma greater than 5 mm in diameter with thickened processes emanating from the soma (*white arrowheads*). For size comparison, the SNpc DA neurons are marked by white asterisks. (**J**) Appearance of activated microglia in the dorsolateral striatum. Microglia in the striatum are slightly larger than in the SNpc. Scale bars: 10 mm (G–J). Statistical analysis was performed using analysis of variance with post hoc tests (Tukey) (Prism 9.0; GraphPad Software) if overall significance was achieved.

In the SNpc, infection with SARS‐CoV‐2 (with or without MPTP) resulted in no change from control animals in the total number of microglia in any of the three experimental groups (F_3,20_ = 0.5944; *P* < 0.626) (Fig. 3A). However, when we quantified resting (Fig. 3B,G) versus activated (Fig. 3C,H) microglia individually, we observed significant differences between groups. Overall differences were seen in both the number of resting microglia (F_3,19_ = 3.572; *P* < 0.033) and activated microglia (F_3,19_ = 7.109; *P* < 0.002).

In regard to resting microglia, we measured a significant 36% reduction in the SARS‐CoV‐2 + MPTP group compared with the vehicle, SARS‐CoV‐2 alone, or MPTP alone (*P* < 0.05). Quantitation of active microglia showed that SARS‐CoV‐2 infection or MPTP alone did not induce a significant increase compared with vehicle (Fig. 3B). We observed a 308% (*P* < 0.002) increase in the SARS‐CoV‐2 + MPTP group compared with vehicle and a 232% increase compared with SARS‐CoV‐2 alone (*P* < 0.006) (Fig. 3C).

In the dorsolateral striatum, infection with SARS‐CoV‐2 (with or without MPTP) resulted in no change in the density of microglia between the control animals and any of the three experimental groups (F_3,20_ = 2.918; *P* < 0.0595) (Fig. 3D). When examining resting versus activated microglia, no change in density was observed in the resting microglia (F_3,20_ = 2.228; *P* < 0.1163) (Fig. 3E,I). However, we did measure a significant change in the density of activated microglia between the groups (F_3,20_ = 9.346; *P* < 0.0005) (Fig. 3F,J). Administration of SARS‐CoV‐2 did not induce a change in activated microglia. However, MPTP alone resulted in a significant 112% increase in the density of activated microglia compared with vehicle (*P* < 0.029). SARS‐CoV‐2 + MPTP induced a 179% increase compared with vehicle (*P* < 0.0003) and a 72% increase compared with SARS‐CoV‐2 alone (*P* < 0.0119).

## Discussion

Through 2021, approximately 300 million people have been infected with SARS‐CoV‐2, of which fewer than 2% have died of the disease.^8^ The majority of these cases involve the original strain isolated in 2020 (USA‐1, also known as alpha; strain B.1.1.7), although the recent variants, delta (strain B.1.617.2) and omicron (strain B.1.1.529), have become the dominant infective strain(s). Although the predominant symptoms associated with this virus are respiratory, it has also been shown that a significant proportion, estimated at 35–40%, also demonstrate neurological sequalae,^38^ including anosmia, headache, seizures, stroke, meningitis, and acute disseminated encephalomyelitis. In addition, a subset of these infected individuals manifests neurological symptoms that appear to have a protracted course, that is, “long haulers.” These patients describe issues related to confusion or “brain fog,” persistent headache, numbness/tingling, loss of sense of small/taste, dizziness, and blurred vision.^39^ Related to longer‐term issues in the post‐COVID‐19 infection period, one also needs to remain cognizant of the possibility for the development of postencephalic syndromes that are known to occur after pandemic viral outbreaks.^40^ Perhaps the most famous of these is the development of both an immediate and a postencephalic parkinsonism that occurred subsequent to the 1918 influenza.^3^, ^41^

Given recent reports of COVID‐19‐induced parkinsonism,^15^, ^16^, ^17^ where the parkinsonian symptoms appear similar to those described after the 1918 H1N1 outbreak, it would be derelict not to consider that these two vastly different pandemic viruses may share a common mechanism that leads to neurological sequalae. Both influenza and SARS‐CoV‐2 are respiratory viruses, both infect epithelial cells in the lung and gut,^42^ and both the 1918 H1N1 influenza virus^43^ and SARS‐CoV‐2^44^ do not appear to have significant inherent neurotropic potential. Both the 1918 influenza and SARS‐CoV‐2 appear to induce an enhanced program of induction of proinflammatory cytokines and chemokines, known as a cytokine storm.^45^ These circulating peripheral cytokines and chemokines can easily penetrate the BBB through capillary beds, as well as communicate with brain parenchyma through brain glymphatics.^46^ Once these inflammatory proteins are in the brain, they have been shown to activate the innate immune system of the brain (microglia and astrocytes), which also begin to secrete inflammatory proteins that have been shown to sensitize neurons to later insults.^47^

The cellular composition of the SNpc, which is the main CNS region that degenerates in PD, is unique in that it contains the highest ratio of microglia/neurons within the CNS. This skewed microglia/neuron ratio places the SNpc at a higher risk for reactive oxygen–induced damage^48^ and disruption of mitochondrial function.^49^ This cellular damage does not necessarily lead to an immediate effect but can result in neurons having a long‐term diminished capacity to handle insults, that is, the “hit and run” effect. This would then lead cells to have a lower threshold for survival after future insults that could include any other agent/environmental^50^ exposure or even genetic sensitivity^51^ associated with PD.

In this study, we modeled in mice genetically modified to express the human ACE2 receptor necessary for infection with SARS‐CoV‐2, recovery from a moderate COVID‐19 infection, and later subthreshold mitochondrial inhibition. Our findings that SARS‐CoV‐2 infection alone did not induce CNS inflammation or SNpc neuron death suggests that this virus, without any other pathology that reduces BBB break, is not a direct parkinsonian agent. However, we do find that systemic infection appears to sensitize the SNpc DA neurons to mitochondrial stress that, in and of itself, does not induce neuron loss. This sensitization appears to remain for a period of time after resolution of the infection and without any apparent physical manifestation of a direct viral inflammation effect in the SNpc. This postinfection sensitization of the SNpc DA neurons is similar to previous studies that have investigated other viruses associated with postencephalic parkinsonism, including the 2009 H1N1 influenza virus pandemic.^27^ However, in this influenza study, the dose of MPTP needed was double that required in this study (4 × 20 mg/kg × 4), and only at this increased dose did they note an increased (21%) SNpc DA neurons loss. In fact, when the animals previously infected with CA/09 H1N1 were administered the same dose of MPTP used in this study (10 mg/kg × 4), no synergistic effects were seen (Supporting Information Fig. S1). This suggests that although different viruses can sensitize the brain to later insults, the dose of SARS‐CoV‐2 virus used here (4 × 10^3^ TCID_50_) is a stronger sensitizing agent than the CA/09 H1N1 influenza virus.

Although these preclinical models of infection leading to later parkinsonism are critical to our understanding of potential downstream outcomes of pandemics, one must also validate the use of our preclinical model. Studies that have examined the cytokine responses in K18‐hACE2 mice and humans after infection with SARS‐CoV‐2 show a similar cell‐type infectivity and a similar induction of cytokine/chemokines.^52^ In both mice and humans, the period of infectivity is similar. In addition, using a similar approach to this study, we previously showed that mice that recovered from an H1N1 influenza infection developed enhanced susceptibility to the parkinsonian toxin MPTP.^53^ The role of H1N1 as a susceptibility agent has been validated in a retrospective study examining risk for developing PD in humans surviving influenza.^54^ This study showed that previous influenza infection resulted in a 73% increased risk for developing PD compared with individuals not infected.^54^ This increased susceptibility was within the confidence interval determined epidemiologically for people born during the time of the 1918 H1N1 pandemic.^55^ Our preclinical studies examining SARS‐CoV‐2 infection suggest the possibility of a similar transient increase in parkinsonian incidence with an additional caveat that our preclinical studies using SARS‐CoV‐2 increased the susceptibility of SNpc DA neurons to low‐dose mitochondrial stress compared with H1N1. Thus, should the predicted risk from SARS‐CoV‐2 manifest, the diverse consequences would represent a substantial burden on patients, families, and society.

Understanding this risk should be a priority. Evaluation of the sensitivity of this mechanism to viral load and heterogeneity across new and emerging variants are important. Although diverse environmental agents have been associated with PD risk, characterizing the effects of the “second hit” across the range of environmental agents^56^, ^57^ beyond the mitochondrial complex I and IV inhibitor used in this study will be necessary. We also need to examine whether SARS‐CoV‐2 infection, in the absence of a direct BBB breach, increases permeability of this barrier that could, in theory, allow greater access to both environmental agents and immune cells into the brain. Another avenue of continued interest is to determine whether treatments for COVID‐19 infection can moderate this viral sensitization. Previously we have shown that prior vaccination against H1N1 or immediate treatment with oseltamivir phosphate can eliminate the H1N1 + MPTP synergy.^53^ Thus, vaccination and antiviral therapies directed to COVID‐19 have the potential to modify our observed increased sensitivity to MPTP (and by inference other environmental agents). However, for the more than 100 million people worldwide who survived COVID‐19, without the benefit of access to vaccinations, the long‐term consequences of infection, including increasing the risk for developing PD, need to be understood. It is also critical for our healthcare providers and governmental agencies to prepare for this potential.

## Author Roles

R.J.S. designed the study, performed experiments, analyzed data, and wrote the paper.

J.B.E. designed experiments, performed experiments, and analyzed data.

D.C. performed experiments and analyzed data.

D.P.O. performed experiments and analyzed data.

M.B. performed experiments.

S.M.A. performed experiments.

S.S. performed experiments.

P.S. designed the study, analyzed data, wrote the paper, and arranged funding for the study.

## Financial Disclosures of All Authors

Stock Ownership in medically related fields: None.

Intellectual Property Rights: None.

Consultancies: D.P.O.: Council member emeritus for Association for Assessment and Accreditation of Laboratory Animal Care (AAALAC) International.

Expert Testimony: None.

Advisory Boards: R.J.S.: Chair of the Scientific Advisory Board of the Parkinson's Foundation; D.P.O.: North Carolina Association for Biomedical Research (BOD) and Sylvan Heights Waterfowl Park (BOD); S.M.A.: Scientific Reports.

Employment: R.J.S., D.C., and M.B.: Thomas Jefferson University; J.B.E., S.M.A., S.S., D.P.O., and P.S.: East Carolina University.

Partnerships: None.

Inventions: None.

Contracts: None.

Honoraria: D.P.O.: AAALAC.

Royalties: None.

Patents: Cells that lack p19INK4D and p27KIP1 activity and methods of use thereof, US Patent 6,589,505, July 8, 2003; Method for Detection of Adenosine and Metabolite Thereof, US Patent 9,528,976, December 27, 2016 (R.J.S.).

Grants: National Institutes of Health: R01NS110084 and R21NS122280; National Institute of Environmental Health Sciences: R01ES030937 (to R.J.S.) and R01HL153115 (to S.S.); State of North Carolina (to R.J.S., J.B.E., S.M.A., S.S., D.P.O., P.S.).

Other: None.

## Supporting information


**Supplemental Figure 1** Comparison of 10 mg/kg MPTP effects between SARS‐CoV‐2 and CA/09 H1N1. 6–8 month old mice were intranasally infected with either 25 μl 4 x 10^3^ TCID_50_ SARS‐CoV‐2 (USA‐1) or 25 μl 10^2^ TCID_50_ A/H1N1/CA/04/2009 (CA/09 H1N1). 45 days after SARS‐CoV‐2 or 30 days after CA/09 H1N1 mice were administered ip 4 x 10 mg/kg MPTP. Seven days after MPTP mice were sacrificed, processed for TH immunohistochemistry and SNpc DA neurons were stereologically assessed. The data for the SARS‐CoV‐2 is identical to that presented in Figure 2A, although individual values are shown. These are used to compare to data from a separate cohort of animals that were intranasally infected with CA/09 H1N1 (n = 14), 4 x 10 mg/kg MPTP (n = 5) or H1N1 + MPTP (n = 4). Unlike the synergy seen following SARS‐C0V‐2, H1N1 did not increase the sensitivity to 4 x 10 mg/kg MPTP to SNpc DA neuron loss. * = p < 0.05 compared to vehicle, SARS‐CoV‐2 + vehicle and MPTP + vehicle, # p < 05 compared to SARS‐CoV‐2 + MPTP.

## Data Availability

Data available on request from the authors
