# Supplementary material for: COVID‐19 Infection Enhances Susceptibility to Oxidative Stress–Induced Parkinsonism
Source: Mov Disord. 2022 Jun 9;37(7):1394–404. doi: 10.1002/mds.29116 (PMC9347874; doi:10.1002/mds.29116)
Supplement: Supplementary file 1 — Supplemental Figure 1 Comparison of 10 mg/kg MPTP effects between SARS‐CoV‐2 and CA/09 H1N1. 6–8 month old mice were intranasally infected with either 25 μl 4 x 10^3^ TCID50 SARS‐CoV‐2 (USA‐1) or 25 μl 102 TCID50 A/H1N1/CA/04/2009 (CA/09 H1N1). 45 days after SARS‐CoV‐2 or 30 days after CA/09 H1N1 mice were administered ip 4 x 10 mg/kg MPTP. Seven days after MPTP mice were sacrificed, processed for TH immunohistochemistry and SNpc DA neurons were stereologically assessed. The data for the SARS‐CoV‐2 is identical to that presented in Figure 2A, although individual values are shown. These are used to compare to data from a separate cohort of animals that were intranasally infected with CA/09 H1N1 (n = 14), 4 x 10 mg/kg MPTP (n = 5) or H1N1 + MPTP (n = 4). Unlike the synergy seen following SARS‐C0V‐2, H1N1 did not increase the sensitivity to 4 x 10 mg/kg MPTP to SNpc DA neuron loss. * = p < 0.05 compared to vehicle, SARS‐CoV‐2 + vehicle and MPTP + vehicle, # p < 05 compared to SARS‐CoV‐2 + MPTP. [file MDS-37-1394-s001.pdf]

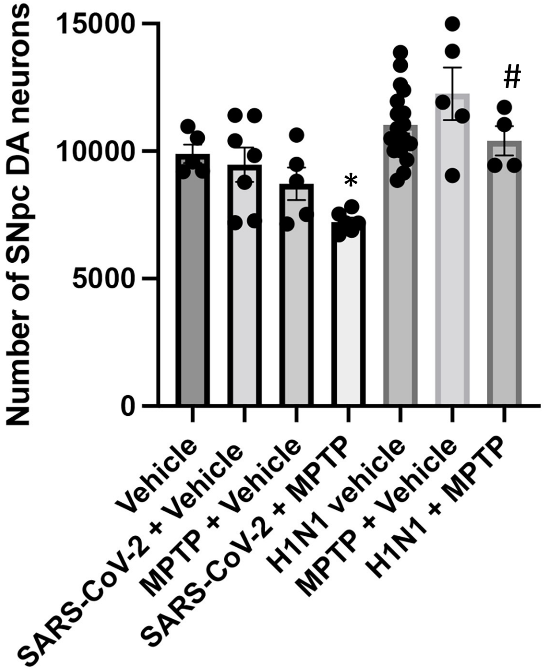

Supplemental Figure 1. Comparison of 10mg/kg MPTP effects between SARS-CoV-2 and CA/09 H1N1. 6-8 month old mice were intranasally infected with either 25 ml 4 x 10^3 $TCID_{50}$ SARS-CoV-2 (USA-1) or 25 ml 10^2 $TCID_{50}$ A/H1N1/CA/04/2009 (CA/09 H1N1). 45 days after SARS-CoV-2 or 30 days after CA/09 H1N1 mice were administered ip 4 x 10 mg/kg MPTP. Seven days after MPTP mice were sacrificed, processed for TH immunohistochemistry and SNpc DA neurons were stereologically assessed. The data for the SARS-CoV-2 is identical to that presented in Figure 2A, although individual values are shown. These are used to compare to data from a separate cohort of animals that were intranasally infected with CA/09 H1N1 (n=14), 4 x 10 mg/kg MPTP (n=5) or H1N1 + MPTP (n=4). Unlike the synergy seen following SARS-C0V-2, H1N1 did not increase the sensitivity to 4 x 10mg/kg MPTP to SNpc DA neuron loss. *= $p<0.05$ compared to vehicle, SARS-CoV-2 + vehicle and MPTP + vehicle, # p<05 compared to SARS-CoV-2 + MPTP.
